# Exploring the burden and support needs of informal caregivers for the older adults in Kazakhstan: a mixed-methods study protocol

**DOI:** 10.3389/fpubh.2023.1248104

**Published:** 2024-01-05

**Authors:** Aliya Zhylkybekova, Andrej M. Grjibovski, Natalya Glushkova, Gulbakit K. Koshmaganbetova

**Affiliations:** ^1^Department of Evidence-Based Medicine and Scientific Management, West Kazakhstan Marat Ospanov Medical University, Aktobe, Kazakhstan; ^2^Central Scientific Research Laboratory, Northern State Medical University, Arkhangelsk, Russia; ^3^Department of Epidemiology and Modern Vaccination Technologies, I.M. Sechenov First Moscow State Medical University (Sechenov University), Moscow, Russia; ^4^Department of Biology, Ecology and Biotechnology, Northern (Arctic) Federal University, Arkhangelsk, Russia; ^5^Department of Epidemiology, Biostatistics and Evidence Based Medicine, Al-Farabi Kazakh National University, Almaty, Kazakhstan

**Keywords:** Kazakhstan, caregivers, aged, caregiver burdens, quality of life

## Abstract

**Background:**

The growing population of older adults, often affected by chronic illnesses, disabilities, or frailty has led to a substantial increase in the need for informal caregivers.

**Objective:**

This paper is a protocol for a study that aims to investigate the effects of caregiving on informal caregivers of older adults in Kazakhstan with special emphasis on the cultural context.

**Methods:**

The protocol outlines a mixed-methods study that will be conducted in four cities in Kazakhstan. A total of 400 informal caregivers of older adults with two or more limitations in Activities of Daily Living (ADL) will be recruited to participate in a survey, aiming to evaluate care-related burdens and quality of life and health-related quality of life. The Institute for Medical Technology Assessment (iMTA) Valuation of Informal Care Questionnaire (iVICQ) was selected to be the main research instrument. Additionally, a subset of participants who express their willingness to participate will be selected from the pool of survey respondents to engage in semi-structured interviews, allowing for a deeper understanding of their experiences and providing insights into their social and medical support needs.

**Conclusion:**

This study will be the first investigation of the impact of caregiving on informal caregivers of older adults in Central Asia. The results will contribute to the literature by providing insights into older adults care within the specific national and cultural context of Kazakhstan with potential generalization to other Central Asian republics of the former USSR.

## 1 Introduction

The latter half of the twentieth century witnessed a significant increase in the global population of older adults (1). According to the projections by the United Nations, it is anticipated that by 2050, the number of older individuals will be twice that of children under five, and will even surpass the working-age population (2). Moreover, it is projected that the average life expectancy will increase to 77.1 years by the year 2050 (2).

Kazakhstan, like many other countries, is also experiencing these demographic trends. The United Nations Population Fund (UNFPA) projects an increase in the number of individuals aged 65 and above from 1.4 million in 2019 to 3.4 million by 2050 (3). Culturally, approximately 64% of older adults in Kazakhstan reside in the household of their family members or relatives, thereby relying on their children or relatives for support (4). The growing number of older adults with chronic diseases, disabilities, or frailty highlights the pressing need for physical support and both formal and informal care.

Engaging in a wide range of caregiving responsibilities can have adverse effects on the health and social well-being of informal caregivers. Moreover, it can result in an increased burden and a reduced quality of life among these individuals. Research findings indicate that caregiver’s own health (5, 6), behavioral disorders of the care recipients, dementia (7), functional dependency of the older adults (8), female gender (9, 10), low education (11), depression, social isolation (12, 13), financial stress (14), lack of care choices (15), co-residence with the recipient of care, and the number of hours dedicated to caregiving (12) are all risk factors that can contribute to the burden experienced by informal caregivers. It is important to consider these factors in order to gain a better understanding of the challenges faced by caregivers who provide informal care with the further going aim to address these challenges and provide appropriate support to caregivers.

Numerous questionnaires have been developed to assess the burden experienced by caregivers. Some of these questionnaires focus on exploring negative effects of caregiving on informal caregivers, while others include items that investigate the positive influence of caregiving. Moreover, the Caregiver Strain Index + (CSI+) questionnaires incorporate both positive and negative inquiring related to caregiving. Table 1 provides examples of the questionnaires most commonly used in the literature.

**Table 1:** Characteristics of questionnaires for measuring caregiver burden.

Questionnaire	Number of items	Scoring scale	Character of questions	Interpretation
Caregiver Burden Inventory (CBI)	24	0–5 Likert Scale	Negative	Score ≤ 36 indicate a risk of a burnout Score ≥ 24 indicate the need to seek some form of respite care
Zarit Burden Interview (ZBI)	22	0–4 Likert Scale	Negative	Score range of 61–88 indicate a risk of burden
Caregiver Strain Index (CSI)	13	Yes/No responses	Negative	Score ≤ 7 indicate a risk of extremely burden
Caregiver Strain Index plus (CSI+)	18	Yes/No responses	Negative & Positive	Score ≤ 7 indicate a risk of extremely burden
Positive Aspects of Caregiving (PAC)	9	0–5 Likert Scale	Positive	Higher scores mean a more positive perception of the caregiving experience

In cultures where there is a strong emphasis on reverence and care for parents or older family members, along with a belief in filial responsibility to provide for their needs, caregiving may not be perceived as highly stressful for caregivers (16, 17). However, it is important to acknowledge that these cultural norms, when combined with family expectations and an increased sense of responsibility, can also be stressful for caregivers and even contribute to an elevated risk of experiencing symptoms of depression (18). Therefore, it is important to assess both the positive and negative effects of caregiving within the context of cultural traditions. In adherence to Kazakhstani traditions, the approach to caring for older adults family members is significantly influenced by cultural values and familial structures. Within this paradigm, it is customary for older adults individuals to cohabit with their children, emphasizing a substantial focus on respecting and fulfilling a sense of duty towards older family members. Considering the cultural idiosyncrasies of Kazakhstan, we posit that individuals engaged in caregiving will demonstrate a heightened occurrence of positive dimensions and derive increased gratification from their caregiving role. Consequently, we anticipate an amelioration in their quality of life, accompanied by a concurrent alleviation of the caregiving burden.

The inclusion of the “Life Course Perspective” and “Stress Process Framework” is essential for understanding and analyzing the caregiving experience. The former considers long-term influences, while the latter examines short-term stressors on caregivers’ well-being. Combining these frameworks in our article helps conceptualize caregiving as a complex process, encompassing both long-term and short-term effects, enduring trends, and crisis moments. This deepens our understanding of how factors shape the caregiving experience, informing the development of effective approaches to support caregivers and enhance their quality of life (19).

Despite the global focus on promoting active longevity among older adults, there is a growing recognition of the urgent need to provide comprehensive support and care for the caregivers involved in their well-being. Kazakhstan has not yet adequately addressed the needs faced by caregivers. The burden they carry remains largely unassessed, with healthcare and social services lacking the necessary tools to evaluate their needs. Moreover, the absence of caregiver registration mechanisms has led to a scarcity of data on the exact number of family caregivers. Therefore, research is warranted to gain a deeper understanding of the impact of caregiving on the burden and quality of life of caregivers in Kazakhstan and other former Soviet republics of Central Asia.

We have developed a research protocol for a study aimed at investigating the impact of caregiving on informal caregivers of older adults in Kazakhstan with special emphasis on the cultural context. Moreover, the study will examine the factors that influence the burden of care, care-related quality of life, and overall health and well-being of caregivers to provide the basis for development of models for medical and social care for caregivers in Kazakhstan.

## 2 Materials and methods

### 2.1 Study design

**Figure 1:**
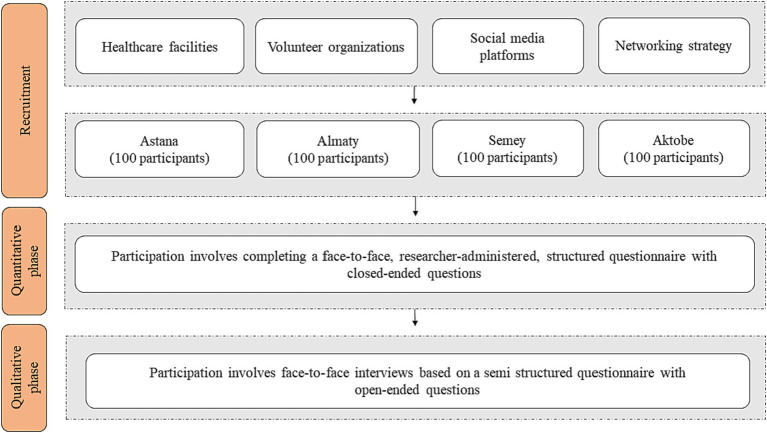
Study plan.

A sequential explanatory mixed-method design will be employed (20). The study will be carried out in two phases across four major cities in Kazakhstan: Astana (the capital), Almaty (the former capital), Semey in the East and Aktobe in the West of the country. The population sizes of these cities are 1.2 million, 2.0 million, 0.8 million and 0.9 million, respectively. The complete study plan is illustrated in Figure 1.

The study will be conducted in two phases. Phase one will consist of a quantitative study, followed by phase two qualitative interviews to provide a more comprehensive understanding of the study results. To collect quantitative data, we will utilize the Institute for Medical Technology Assessment (iMTA) Valuation of Informal Care Questionnaire (iVICQ) (21) as the research instrument. This questionnaire will primarily focus on assessing the levels of burden associated with caregiving, as well as the quality of life and health-related quality of life of the participants.

In phase two, the focus will be on collecting qualitative data to explore the contextual aspects of caregiving including experiences, needs, and requirements of caregivers in relation to providing care, as well as the availability of healthcare-and social support. To gather this information, we will conduct semi-structured interviews with participants selected from the pool of respondents until we reach saturation. The interviews will be analyzed using an inductive content analysis approach to get insights into the caregivers’ experiences (22).

### 2.2 Sample size

For the quantitative phase, we will invite 400 informal caregivers from four cities in Kazakhstan, with 100 informal caregivers selected from each city, to participate in the study. The sample size was calculated to assess the prevalence of binary outcomes with a precision of 5% for an infinite population. The calculated minimal sample size was 396, which we rounded up to 400. As for informal caregivers, we were more interested in associations between numeric outcomes and potential predictions, therefore we calculated sample size required for applying linear regression models with 5 predictors to explain at least 15% of the total variance with levels of alpha-and beta errors of 5 and 20%, respectively. All calculations were made with G-Power software. Only primary caregivers will be invited. However, in cases where two caregivers caring for the same older adult identify themselves as primary caregivers, we will include both of them.

During the qualitative phase, participants who completed the questionnaire will be invited to participate in interviews. Subsequently, the recordings of each interview will be encoded. The data collection process will continue until the saturation point is reached (22). After reaching this point, additional 2-3 interviews will be conducted to ensure that no further data collection provides new information or leads.

### 2.3 Eligibility criteria

All individuals who will give their consent to participate and meet the eligibility criteria will be included in the study. The inclusion and exclusion criteria for family caregivers are presented in Table 2.

**Table 2:** Eligibility criteria.

Inclusion	Exclusion
≥18 years of age	Cognitive impairment or mental illness
Experience of providing informal care for older adults aged ≥ 65 years	No informed consent
Older adults should have at least two limitations in activities of daily living.	
Provision of informal care for ≥ 2 weeks	
Proficiency in either Kazakh or Russian	
Permanent residence in Kazakhstan	

### 2.4 Recruitment of participants

As there is no national registry of informal caregivers in Kazakhstan, a diverse range of recruitment strategies will be employed to ensure representativeness of the sample. The following approaches will be utilized:

- Recruitment via healthcare facilities: we will identify older adults aged 65 and above who face limitations in their daily activities through healthcare facilities in the four study sites. Subsequently, we will reach out to family caregivers either through phone calls or home visits to extend an invitation for their participation in the survey.
- Recruitment via volunteer organizations: we will partner with volunteer organizations that offer support to individuals facing challenging circumstances. These organizations will serve as platforms to reach out to potential caregivers.
- Advertising on social media platforms: we will advertise the study across multiple social media platforms, targeting individuals who may be fulfilling caregiving roles for older adults. By using the power of social media, we aim to maximize the visibility of the study and attract potential participants.
- To enhance the efficiency of recruitment, we will implement a networking strategy will be employed. We will actively encourage participants who have already agreed to take part in the study will be encouraged to refer other eligible individuals from their social networks who might be interested in participating in the study.

Upon agreeing to participate, caregivers will be requested to provide informed consent by signing a consent form. Confidentiality and anonymity of all participants will be ensured.

### 2.5 Data collection

#### 2.5.1 Phase one

In the quantitative phase, we will be using the iVICQ questionnaire (21) as a comprehensive, consistent, validated, and non-disease-specific toolkit for investigating informal care. This questionnaire is not limited to a specific disease. It allows for the collection of information about both caregivers and care recipients. Additionally, it includes tools for assessing the caregiving burden, quality of life, and health-related quality of life. It can be used the entire questionnaire or specific sections, making it suitable for quick screening using brief scales. To ensure linguistic accuracy and cultural appropriateness, we have conducted a rigorous process of independent forward and backward translations for the Kazakh and Russian versions of the iVICQ questionnaire. Native language researchers proficient in English, Kazakh, and Russian reviewed the translations for accuracy. The survey questionnaire will be pilot tested in the study sites prior to the main study.

The questionnaire will be administered in paper format by members of the research team. It is expected that participants will need approximately 20–25 min to complete the questionnaire. The opinions of informal caregivers will be used to collect the characteristics of care recipients.

The primary section of the questionnaire focuses on gathering demographic characteristics about informal caregivers and care recipients. This includes date of birth, gender, education level, family income, and marital status. Furthermore, we will assess the health-related quality of life using the EQ-5 L-5D questionnaire (23). To assess the functioning of care recipients, we will use the Barthel index (24). Additionally, it includes characteristics of the informal care situation, such as the number of hours per week dedicated to caregiving, the intensity of informal care, and the need for professional assistance.

The second section focuses on assessing the subjective burden and evaluating the well-being of caregivers using the Caregiver Strain Index Plus (CSI+) (25), the Care-related Quality of Life Instrument (CarerQol-7D) (26), the Self-Rated Burden scale (SRB) (27), the Assessment of Informal Care Situation (ASIS) (28), Process Utility (PU) (27), and Perseverance time (Pt) (29). Furthermore, the monetary valuation of informal care will be determined using the opportunity cost method (30). A summary of these variables and the recoding process is presented in Table S1 in the Supplementary material.

In accordance with the classification of education levels, we will utilize the nine-level classification system employed in the Kazakhstan National Census of 2020 (31). The education levels will be categorized into three main categories: low, medium, and high, following the International Standard Classification of Education. The low category represents education levels below secondary or equivalent, while the high category denotes university or higher education level (32).

To classify income data, we will adopt the classification methodology used in the Kazakhstan National Census of 2020 (31). This methodology takes into account small gaps between income levels to ensure accurate representation of the caregiver’s income (33). The income data will be divided into three categories: low, medium, and high. The low category represents income levels below the average wage, while high signifies an income higher than the average wage in Kazakhstan.

To assess the health-related quality of life, we will apply the EQ-5D-5L questionnaire. To ensure linguistic accuracy, we will utilize the available interviewer administration version of the EQ-5D-5L questionnaire in both Kazakh and Russian languages. These versions have been translated and certified by the Legal EuroQol team, ensuring the accuracy and reliability of the translation.

#### 2.5.2 Phase two

Upon completing of the survey, participants will be invited to participate in a qualitative study. Face-to-face interviews based on a semi-structured questionnaire comprising open-ended questions will be conducted at a time and location convenient for the participants. The interview guide will cover specific topics while allowing for adaptability and flexibility to accommodate the unique experiences and concerns of participants.

Prior to the interview, participants will receive a comprehensive overview of the study objectives and framework. The interviews will be recorded. Each interview is expected to last approximately 30-45 min. The guide for conducting semi-structured interviews is presented in Table S2 in the Supplementary material.

### 2.6 Data analysis

#### 2.6.1 Phase one

The quantitative data analysis will be performed using R-Studio Version R4.1.3 (2009–2023 Posit Software, PBC). Distributions of continuous variables and frequencies of categorical variables will be assessed followed by calculations of descriptive statistics. Normally distributed numeric data will be presented using means and standard deviations. Medians and quartiles will be used for skewed data. Chi-squared tests will be utilized for bivariate analysis of categorical data. Categorical data will be presented as proportions with 95% confidence intervals (CI). To identify independent predictors of care-related burden we will use multivariable logistic regression while for quality of life, we will apply multivariable linear regression.

#### 2.6.2 Phase two

In the analysis of qualitative data, descriptive statistics will be utilized to summarize demographic information. The interviews will be transcribed verbatim, capturing all spoken words. For text analysis, we will utilize MAXQDA 2022 software. Semi-structured interviews will be conducted, applying an inductive content analysis approach to analyze the participants’ experiences. The transcribed texts will be divided into meaningful units, known as semantic units, which will be assigned codes while preserving the contextual information. These codes will then be analyzed and organized into relevant categories based on their similarities and connections. Throughout this process, similar categories will be integrated to form subthemes, ultimately leading to the emergence of main themes.

## 3 Discussion

The proposed study represents a pioneering effort to investigate the impact of caregiving on informal caregivers for older adults in Kazakhstan. The study employs a mix-method approach to facilitate a comprehensive examination of informal caregiving in this Central Asian republic. The quantitative component will assess the care related burden and quality of life among caregivers. The qualitative component will gather valuable insights into caregivers’ experiences, needs, and requirements. The advantages of mixed design research include providing a new understanding of the complexity and multifaceted nature of healthcare research. By integrating both quantitative and qualitative methods, we can optimize the breadth and depth of the research. This approach will allow us not only to consider the socio-cultural context but also the intricacies of caregiving that closely resemble real-life situations. By collecting comprehensive data through mixed studies, we can gain a deeper understanding of the problem at hand and explore potential solutions (20, 34).

This research protocol is designed to examine the quality of life and caregiving burden associated with informal care, as well as to identify the most significant predictors and needs within the understudied population of informal caregivers in Kazakhstan with potential implications for other countries of Central Asia. The study focuses on a broad range of informal caregivers providing care in the context of disability, illness, aging, or frailty.

The burden of caregiving can vary depending on the specific condition (35–40). However, solely focusing on particular illnesses restricts the identification of common determinants of caregiver burden for older individuals (41, 42). Understanding the overarching determinants can help in the development of effective policies and interventions that promote health and well-being. Therefore, it is essential to develop specific interventions that aim to enhance the physical and mental independence of patients, while also addressing the social support needs of caregivers (42). These interventions should be targeted towards individuals who are at the highest risk and actively engaged in caregiving.

Despite the commonality of caregiver needs, research indicates that there are variations in the well-being of caregivers across different countries (43). This implies that caregiver needs differ depending on the country of residence and specific cultural and socio-economic conditions. Therefore, there is a need for studies that investigate the needs of informal caregivers in specific countries, considering demographic, cultural, and economic factors (45–47).

In many Asian countries, deeply ingrained and sometimes explicit legislative norms dictate that the responsibility of caring for older adults family members falls on children and relatives. An exceptional aspect of Asian and particularly Central Asian culture is the practice of multigenerational households, where several generations including grandparents, parents, children, and grandchildren live together (17, 48, 49). This arrangement often leads to the delegation of caregiving duties to family members, resulting in an underdeveloped system of social and specialized medical support for caregivers (50). The research on caregiving burden in Asian countries yields conflicting findings. Some studies suggest that the concept of filial responsibility can mitigate the burden experienced by individuals (18). Conversely, other studies propose that such responsibilities may lead to increased stress and burden (51). Given these disparities, there is a need for a study focused on the caregiving burden in Central Asian countries. The findings from such a study would significantly contribute to shaping and enhancing social support systems for caregivers in the Central Asian region.

Caring responsibilities significantly have a substantial impact on the quality of life for caregivers. Specifically, caregivers often experience a higher frequency of physical and health-related issues, resulting in a decline in their perceived quality of life (52–54). However, there is evidence to suggest that psychosocial interventions can alleviate the burden on caregivers and improve their quality of life (55).

The utilization of tools to assess the impact on caregivers is important, as these instruments can be employed in primary healthcare settings as frontline operational tools (56). This allows for the identification and timely referral of caregivers to healthcare and social service specialists, reducing the burden on caregivers and enabling them to provide care for older individuals at home for an extended period.

As the demand for informal caregiving continues to rise, our study will document the experiences and needs of home-based caregivers. Conducting an in-depth analysis of this field will be valuable in fostering the improvement of care for older individuals taking into account the needs of both caregivers and care recipients.

The caregiving situation for informal caregivers can vary depending on the relationships they have with care recipients, such as being a spouse or adult child. It is possible that these different informal caregivers also have distinct needs. In a systematic review of mixed studies (57) aimed at examining and comparing the needs of different groups of informal caregivers based on their relationships with care recipients, the results showed that, alongside common needs, the studied groups also had unique needs. The synthesis of the 22 included articles led to the identification of seven themes of needs among spouses, adult children, and adult siblings as informal caregivers: the need for information, support, personal time, addressing personal issues such as managing their own health, maintaining their relationship with their care recipient, being recognized and considered as a caregiver. In our study, we will explore and compare the identified needs of different groups of informal caregivers based on their relationships with care recipients. Understanding the needs of different groups of informal caregivers in Kazakhstan can contribute to the development of individualized solutions to enhance their quality of life and that of their care recipients.

### 3.1 Potential limitations of the study

Firstly, since there is no national registry of caregivers in Kazakhstan, it is challenging to obtain a representative sample. However, we used four strategies to improve representativeness of the sample for better generalization of the results.

Secondly, as this is a cross-sectional in nature, it does not allow drawing conclusions on cause-effect relationships. Further longitudinal studies will be required to explore causal effects of the identified associations.

Thirdly, it is possible that we may not be able to capture all perspectives in the qualitative research component due to limitations in the sample size. However, we will strive to delve deeply into the topic during interviews to conduct a detailed analysis of the experiences and needs of caregivers.

In spite of these limitations, this study represents the first attempt in Central Asia in general and in Kazakhstan in particular to utilize CSI+ and CarerQol-7D tools to measure care-related burden and quality of life among informal caregivers.

## Data availability statement

The original contributions presented in the study are included in the article/Supplementary material, further inquiries can be directed to the corresponding author.

## Ethics statement

The study presented in this research protocol will be performed in accordance with Declaration of Helsinki. The study protocol was approved by the Research Ethics Committee of West Kazakhstan Marat Ospanov Medical University, Aktobe, Kazakhstan on November 19, 2021. The anonymity of participants will be guaranteed. No personal data will be utilized in the project.

## Author contributions

AZ, GK, and NG contributed to conception and design of the study. AZ wrote the first draft of the manuscript. AZ, AG, and GK wrote sections of the manuscript. AZ, AG, and NG edited the final version. All authors contributed to the article and approved the submitted version.
