# Supplementary material for: Exploring the burden and support needs of informal caregivers for the older adults in Kazakhstan: a mixed-methods study protocol
Source: Front Public Health. 2024 Jan 5;11:1248104. doi: 10.3389/fpubh.2023.1248104 (PMC10796845; doi:10.3389/fpubh.2023.1248104)
Supplement: Supplementary file 1 [file Table_1.DOCX]

Supplementary Material

**Exploring the burden and support needs of informal caregivers for the older-adult in Kazakhstan: A mixed-methods study protocol**

Aliya Zhylkybekova^1^*, Andrej M. Grjibovski ^2,3,4^ , Natalya Glushkova ^5^, Gulbakit K. Koshmaganbetova^1^

^1^Department of Evidence-Based Medicine and Scientific Management, West Kazakhstan Marat Ospanov Medical University, Aktobe, Kazakhstan

^2^Central Scientific Research Laboratory, Northern State Medical University, Arkhangelsk, Russian Federation

^3^Department of Epidemiology and Modern Vaccination Technologies, I.M. Sechenov First Moscow State Medical University (Sechenov University), Moscow, Russian Federation

^4^Department of Biology, Ecology and Biotechnology, Northern (Arctic) Federal University, Arkhangelsk, Russian Federation

^5^Department of Epidemiology, Biostatistics and Evidence Based Medicine, Al-Farabi Kazakh National University, Almaty, Kazakhstan

*** Correspondence: Aliya Zhylkybekova:** [**zhylkybekovaa@gmail.com**](mailto:zhylkybekovaa@gmail.com)

Table S1. Title: Table S1. Variables, Categories, and Recoding i iVICQ

| **Variables** | **Categories in iVICQ**  **home care** | | **Categories of variables** | **Re-coding** | **Section** |
| --- | --- | --- | --- | --- | --- |
| Background characteristics informal caregiver | | | | | Section C |
| What city/region do you live in? | Astana  Almaty  Semey  Aktobe | | Nominal variable | Re-code to  1=Astana  2=Almaty  3=Semey  4=Aktobe | C1 |
| Domicile code | Urban  Rural | | Binary  variable | Re-code to  1 = urban  2 = rural | C2 |
| Gender | Female  Male | | Binary  variable | Re-code to  1=Female  2=Male | C3 |
| Age |  | | Continuous variable | Categorise continuous variable to  1= 18-24; 2= 25-34; 3= 35-44;  4= 45-54; 5= 55-64 and 6= 65+ | C4 |
| Ethnicity | Kazakh  Russian  Other | | Nominal variable | Re-code to  1 = Kazakh  2= Russian  3= Other | C5 |
| Educational level | Elementary education  Basic secondary education  General secondary education  Primary professional education  Professional secondary education  Professional technical education  Incomplete higher education  University degree  Postgraduate study | | Ordinal  variable | Re-code to  Low  Middle  High | C6 |
| Partner | No  Yes | | Binary  variable | Re-code to  0= No  1= Yes | C7 |
| Children | No  Yes | | Binary  variable | Re-code to  0 = No  1= Yes | C8 |
|  | If yes, write the number of children | | Discrete  variable | No change  ___under 18  ___older than 18 | C 8.1  C8.2 |
| Living with child | No  Yes | | Binary  variable | Re-code to  0 = No  1 = Yes | C9 |
|  | If yes, write the number of children | | Discrete  variable | ___ number | C 9.1 |
| Paid work | No  Yes | | Binary  variable | Re-code to  0 = No  1 = Yes | C10 |
|  | If yes, full time or  part- time | | Binary  variable | Re-code to  0 = Part time  1 = Full time | С 10.1 |
| Unpaid work | No  Yes | | Binary  variable | Re-code to  0 = No  1 = Yes | C 11 |
|  | If yes, write the number | | Continuous variable | __ hours per month | C 11.1 |
| Financial compensation for providing informal care | No  Yes | | Binary  variable | Re-code to  0=No  1=Yes | C 12 |
|  | If yes, write the sum | | Continuous variable | ___ per month | C 12.1 |
| Do you feel you had a choice in taking on this responsibility for caring for your [relation]? | No  Yes | | Binary  variable | Re-code to  0 = No  1 = Yes | С 13 |
| Monthly net household income* | 1. no income  2. less than 10000 KZT  3. from 10001 to 50000 KZT  4. from 50001 to 100000 KZT  5. from 100001 to 150000 KZT  6. from 150001 to 200000 KZT 7. from 200001 to 250000 KZT  8. from 250001 to 300000 KZT  9. from 300001 to 500000 KZT  10. from 500001 to 1000000 KZT  11. more than 1000000 KZT | | Ordinal  variable | Re-code to  Low  Middle  High | C 14 |
| Health EQ-5D | | | | |  |
| Mobility | No problem  Slight problems  Moderate problems  Severe problems  Unable to /extreme problems | | Ordinal  variable | Re-code to  1 = no problem  2 = slight problems  3 = moderate problems  4 = severe problems  5 = unable to /extreme problems | C 15.1 |
| Self-Care |  |  |  |  | C 15.2 |
| Usual activities |  |  |  |  | C 15.3 |
| Pain/Discomfort |  |  |  |  | C 15.4 |
| Anxiety/ Depression |  |  |  |  | C 15.5 |
| Health VAS | From 1 to 100 | | Discrete  variable | No change | C 16 |
| Background characteristics care recipient | | | | | Section D |
| Gender | Female  Male | | Binary  variable | Re-code to  1=Female  2=Male | D1 |
| Age |  | | Continuous variable | Categorise continuous variable to 1= age 65-69; 2 = age 70+, 3 = age 80+ | D2 |
| Health EQ-5D | | | | | |
| Mobility | No problem  Slight problems  Moderate problems  Severe problems  Unable to /extreme problems | | Ordinal  variable | Re-code to  1 = no problem  2 = slight problems  3 = moderate problems  4 = severe problems  5 = unable to  /extreme problems | D3.1 |
| Self-Care |  |  |  |  | D3.2 |
| Usual activities |  |  |  |  | D3.3 |
| Pain/Discomfort |  |  |  |  | D3.4 |
| Anxiety/ Depression |  |  |  |  | D3.5 |
| Health VAS | From 1 to 100 | | Discrete  Variable | No change | D4 |
| Health problem | Temporary disease with the prospect of complete recovery  Chronic disease or disability  Dementia or memory problems  Mental problems  Problems due to aging  Terminal disease | | Nominal  variable | Re-code to  1= Temporary disease  2= Chronic disease  3= Dementia  4= Mental problems  5= Problems due to aging  6 = Terminal disease | D5 |
| Bartel Index for Activities of Daily Living (ADL) | | | | | |
| Feeding | Independed  Needs help  Unable | | Ordinal  variable | Re-code to  10 = In depended  5 = Needs help  0 = Unable | D 6.1 |
| Personal hygiene | Independed  Unable | |  | 5=In depended  0=Unable | D 6.2 |
| Dressing | Independed  Needs help  Unable | |  | 10=Independed  5=Needs help  0=Unable | D 6.3 |
| Bathing | Independed  Unable | |  | 5=Independed  0=Unable | D 6.4 |
| Bowel and Bladder control | Independed  Needs Help  Unable | |  | 20=Independed  10=Needs help  0=Unable | D 6.5 |
| Toilet use | Independed  Needs help  Unable | |  | 10 =Independed  5=Needs help  0=Unable | D 6.6 |
| Transfers (bed to chair and back) | Independed  Needs minor help (verbal or physical)  Needs major help (1-2 people, physical), can sit  Unable | |  | 15=Independed  10=Needs minor help  5=Needs major help  0=Unable | D 6.7 |
| Mobility on level surfaces | Independet (but may use any aid, e.g. stick) >50 yards  Walks with help of one person (verbal or physical) >50 yards  Wheelchair independent, including corners, >50 yards  Immobile or <50 yards | |  | 15=Independed  10=Walks with help  5=Wheelchair independent  0=Immobile | D 6.8 |
| Stairs | Independed  Needs help (verbal, physical, carrying aid)  Unable | |  | 10=Independed  5=Needs help  0=Unable | D 6.9 |
| Relationship between informal caregiver and care recipient | My partner  Mother or father  Mother-in-law or father-in-law  Daughter or son  Another family member  Friend  Acquaintance or neighbour  Other | | Nominal  variable | Re-code to  1= my partner  2= mother or father  3= mother-in-law or father-in-law  4= daughter or son  5= another family member  6 = friend  7 = acquaintance or neighbour  8= other | D7 |
| Characteristics of the informal care situation | | | | | Section E |
| Duration of informal care | Less than month__weeks  Less than year___month  More than year__ years | | Continuous  variable | No change | E1.1  Е1.1  Е1.2 |
| Intensity of informal care | 1 day per week  2 days per week  3 days per week  4 days per week  5 days per week  6 days per week  7 days per week | | Ordinal  variable | No change | E2 |
| Did you spend time during the last week on the following activities in her/his house? | | | | | |
| Preparation of food and drinks? | No  Yes | | Binary  variable | Re-code to  0= No  1=Yes | E3.1 |
|  | If ‘Yes’ how many ___minute in day or  ___hours in week | | Continuous variable | No change | E3.1.1  Е3.1.2 |
| Cleaning the house? | No  Yes | | Binary  variable | 0=No  1=Yes | E3.2 |
|  | If ‘Yes’ how many ___minute in day or  ___hours in week | | Continuous  variable | No change | E3.2.1  Е3.2.2 |
| Washing, ironing and sewing? | No  Yes | | Binary  variable | 0= No  1=Yes | E3.3 |
|  | If ‘Yes’ how many ___minute in day or  ___hours in week | | Continuous variable | No change | E3.3.1  Е3.3.2 |
| Taking care of and playing with your children? | No  Yes | | Binary  variable | 0=No  1=Yes | E3.4 |
|  | If yes, ___minute in day  ___hours in week | | Continuous variable | No change | E3.4.1  Е3.2.2 |
| Shopping? | No  Yes | | Binary  variable | 0=No  1=Yes | E3.5 |
|  | If ‘Yes’ how many ___minute in day or  ___hours in week | | Continuous variablе | No change | E3.5.1  Е3.5.2 |
| Maintenance work, odd jobs, gardening? | No  Yes | | Binary  variable | 0=No  1=Yes | E3.6 |
|  | If ‘Yes’ how many ___minute in day or  ___hours in week | | Continuous variable | No change | E3.6.1  Е3.6.2 |
| Did you spend time during the last week assisting her/him with the activities below? | | | | | |
| Personal care (dressing/undressing, washing, combing, shaving)? | No  Yes | | Binary  variable | 0=No  1=Yes | E3.7 |
|  | If ‘Yes’ how many ___minute in day or  ___hours in week | | Continuous variable | No change | E3.7.1  Е3.7.2 |
| Going to the toilet? | No  Yes | | Binary  variable | 0=No  1=Yes | E3.8 |
|  | If ‘Yes’ how many ___minute in day or  ___hours in week | | Continuous variable | No change | E3.8.1  E3.8.2 |
| Moving around the house? | No  Yes | | Binary  variable | 0=No  1=Yes | E3.9 |
|  | If ‘Yes’ how many ___minute in day or  ___hours in week | | Continuous variable | No change | E3.9.1  Е3.9.2 |
| Eating and drinking? | No  Yes | | Binary  variable | 0=No  1=Yes | E3.10 |
|  | If ‘Yes’ how many ___minute in day or  ___hours in week | | Continuous variable | No change | E3.10.1  Е3.10.2 |
| Mobility outside the house? | No  Yes | | Binary  variable | 0=No  1=Yes | E3.11 |
|  | If ‘Yes’ how many ___minute in day or  ___hours in week | | Continuous  variable | No change | E3.11.1  Е3.11.2 |
| Making trips and visiting family or friends? | No  Yes | | Binary  variable | 0=No  1=Yes | E3.12 |
|  | If ‘Yes’ how many ___minute in day or  ___hours in week | | Continuous  variable | No change | E3.12.1  Е3.12.2 |
| Visiting a doctor or the hospital? | No  Yes | | Binary  variable | 0=No  1=Yes | E3.13 |
|  | If ‘Yes’ how many ___minute in day or  ___hours in week | | Continuous  variable | No change | E3.13.1  Е3.13.2 |
| Organizing help, physical aids or house adaptations? | No  Yes | | Binary  variable | 0=No  1=Yes | E3.14 |
|  | If ‘Yes’ how many ___minute in day or  ___hours in week | | Continuous  variable | No change | E3.14.1  Е3.14.2 |
| Taking care of financial matters like insurance? | No  Yes | | Binary  variable | 0=No  1=Yes | E3.15 |
|  | If ‘Yes’ how many ___minute in day or  ___hours in week | | Continuous  variable | No change | E3.15.1  Е3.15.2 |
| Can she/he be left alone? | No, she/he needs continuous surveillance  Yes, but not for more than one hour  Yes, she/he can easily be left alone for several hours (or more) | | Ordinal  variable | Re-code to  0= No, she/he needs continuous surveillance  1= Yes, but not for more than one hour  2= Yes, she/he can easily be left alone for several hours | E4 |
| Do you share a household with her/him? | No  Yes | | Binary  variable | Re-code to  0=No  1=Yes | E5 |
|  | If ‘No’ how many minutes of travel distance from her/him | | Continuous  variable | No change | E5.1 |
| Does she/he live independently? | No, she/he lives in a residential or nursing home  No, she/he lives in another health institution  Yes, and she/he lives alone  Yes, and she/he shares a household with at least one other person  Other | | Nominal variable | Re-code to  1 = No, she/he lives in a residential or nursing home  2=No, she/he lives in another health institution  3=Yes, and she/he lives alone  4=Yes, and she/he shares a household with at least one other person  5=Other: | E6 |
|  | If ‘Other’______________ | |  | No change | E6.1 |
| Besides your care or support, does she/he also receive care from a professional caregiver at home? | No  No, but she/he is on a waiting list for professional care at home per week  Yes | | Nominal  variable | 1=No  2=No, but she/he is on a waiting list for professional care at home per week  3=Yes | E7 |
| Does she/he visit a day care facility or a residential or nursing home? | No  No, but she/he is on a waiting list for day care  Yes | | Nominal  variable | 1=No  2=No, but she/he is on a waiting list for day care  3=Yes | E8 |
| Does she/he need more professional care than she/he receives at the moment? | No  Yes | | Binary  variable | Re-code to  0=No  1=Yes | E9 |
| Does she/he also receive care from other informal caregivers? | No, I am the only informal caregiver  Yes | | Binary  variable | Re-code to  0=No  1=Yes | E10 |
|  | If ‘Yes’ how many people? | | Discrete  variable | No change | E10.1 |
|  | If ‘Yes’ how many hours in a last week | | Continuous variable | No change | E10.2 |
| Subjective burden, health and well-being (non-monetary valuation) | | | | | Section F |
| Caregiver Strain Index (CSI+) | | | | | |
| Sleep is disturbed | No  Yes | | Binary  variable | Re-code to  0=No  -1=Yes | F1.1 |
| It is inconvenient | No  Yes | |  | Re-code to  0=No  -1=Yes | F1.2 |
| She/he appreciates everything I do for her/him | No  Yes | |  | Re-code to  0=No  -1=Yes | F1.3 |
| It is a physical strain | No  Yes | |  | Re-code to  0=No  -1=Yes | F1.4 |
| It is confining | No  Yes | |  | Re-code to  0=No  -1=Yes | F1.5 |
| Besides the care I provide to her/him, I have enough time for myself | No  Yes | |  | Re-code to  0=No  -1=Yes | F1.6 |
| There have been family adjustments | No  Yes | |  | Re-code to  0=No  -1=Yes | F1.7 |
| There have been changes in personal plans | No  Yes | |  | Re-code to  0=No  -1=Yes | F1.8 |
| There have been other demands on my time | No  Yes | |  | Re-code to  0=No  -1=Yes | F1.9 |
| There have been emotional adjustments | No  Yes | |  | Re-code to  0=No  -1=Yes | F1.10 |
| I can handle the care for her/him fine | No  Yes | |  | Re-code to  0=No  -1=Yes | F1.11 |
| Some behaviour is upsetting | No  Yes | |  | Re-code to  0=No  -1=Yes | F1.12 |
| It is upsetting to find that she/he has changed so much  from her/his former self | No  Yes | |  | Re-code to  0=No  -1=Yes | F1.13 |
| I am happy to care for her/him | No  Yes | |  | Re-code to  0=No  1=Yes | F1.14 |
| There have been work adjustments | No  Yes | |  | Re-code to  0=No  1=Yes | F1.15 |
| Feeling completely overwhelmed | No  Yes | |  | Re-code to  0=No  1=Yes | F1.16 |
| It is a financial strain | No  Yes | |  | Re-code to  0=No  1=Yes | F1.17 |
| Taking care for her/him is important to me | No  Yes | |  | Re-code to  0=No  1=Yes | F1.18 |
| Self-rated burden scale (SRB) | | | | | |
| How burdensome do you feel caring for or accompanying her/him is at the moment? | VAS-scale  From 0 to 10  0 mean not at all straining  10 mean much too straining | | Discrete variable | No change | F2 |
| Perseverance time (Pt) | | | | |  |
| How long will you be able to carry on giving care? | Less than one week  More than one week but less than one month  More than one month but less than six months  More than six months but less than one year  More than one year but less than two years  More than two years | | Ordinal  Variables | Re-code to  1= Less than one week  2= More than one week but less than one month  3= More than one month but less than six months  4= More than six months but less than one year  5= More than one year but less than two years  6= More than two years | F3 |
| Care-related Quality of Life CarerQOl-7D | | | | | |
| I have fulfilment from carrying out my care tasks | No  Some  A lot of | | Nominal variables | Re-code to  0=No  1=Some  2=A lot of | F4.1 |
| I have relational problems with the care receiver | No  Some  A lot of | |  | Re-code to  0=No  1=Some  2=A lot of | F4.2 |
| I have problems with my own mental health | No  Some  A lot of | |  | Re-code to  0=No  1=Some  2=A lot of | F4.3 |
| I have problems combining my care tasks with my own daily activities | No  Some  A lot of | |  | Re-code to  0=No  1=Some  2=A lot of | F4.4 |
| I have financial problems because of my care tasks | No  Some  A lot of | |  | Re-code to  0=No  1=Some  2=A lot of | F4.5 |
| I have support with carrying out my care tasks, when I need it | No  Some  A lot of | |  | Re-code to  0=No  1=Some  2=A lot of | F4.6 |
| I have problems with my own physical health | No  Some  A lot of | |  | Re-code to  0=No  1=Some  2=A lot of | F4.7 |
| Care-related Quality of Life VAS | | | | |  |
| How happy do you feel at the moment? | VAS-scale  From 0 to 10  0 mean completely unhappy  10 mean completely happy | | Discrete variable | No change | F5 |
| Assesment of caregiving situation scale (ASSIS) | | | | | |
| How would you rate your current caregiving situation? | VAS-scale  From 0 to 10  0 mean worst imaginable caregiving situation  10 mean best imaginable caregiving situation | | Discrete variable | No change | F6 |
| Process Utility (PU) | | | | | |
| How happy you would feel if all your caregiving tasks were taken over by this person? | VAS-scale  From 0 to 10  0 mean completely unhappy  10 mean completely happy | | Discrete variable | No change | F7 |
| She/he would have a problem with another person taking over my caregiving tasks. | 7- point Lakert scale  completely agree  agree to a large extent  rather agree  agree nor disagree  rather disagree  disagree to a large extent  completely disagree | | Ordinal  Variable | No change  1 completely agree  2 agree to a large extent  3 rather agree  4 agree nor disagree  5 rather disagree  6 disagree to a large extent  7 completely disagree | F8 |
| I would have a problem with another person taking over my caregiving tasks for her/him | 7- point Lakert scale  completely agree  agree to a large extent  rather agree  agree nor disagree  rather disagree  disagree to a large extent completely disagree | | Ordinal  Variable | No change  1 completely agree  2 agree to a large extent  3 rather agree  4 agree nor disagree  5 rather disagree  6 disagree to a large extent  7 completely disagree | F9 |
| Monetary valuation of informal care | | | | | Section G |
| Opportunity cost method | | | | | |
| Time forgone of paid work due to informal care | No, I did not have paid work before  No, I still perform the same amount of paid work  Yes, I forgone of paid work due to informal care | | Nominal  variable | Re-code to  0= No, I did not have paid work before  1= No, I still perform the same amount of paid work  3= Yes, I forgone of paid work due to informal care | G1 |
|  | If ‘Yes’ for_____(number) fewer hours per week  since ______(year) | | Continuous variable | No change | G1.1  G1.2 |
| Time forgone of unpaid work due to informal care | No, I did not have unpaid work before  No, I still perform the same amount of unpaid work  Yes, I forgone of unpaid work due to informal care | | Nominal  variable | Re-code to  0= No, I did not have unpaid work before  1= No, I still perform the same amount of unpaid work  3= Yes, I forgone of unpaid work due to informal care | G2 |
|  | If ‘Yes’ for _____(number) fewer hours per week  since _______(year) | | Continuous variable | No change | G2.1  G2.2 |
| Time forgone of leisure time due to informal care | No, I did not have leisure time before  No, I still perform the same amount of leisure time  Yes, I forgone of leisure time due to informal care | | Nominal  variable | Re-code to  0= No, I did not have leisure time before  1= No, I still perform the same amount of leisure time  3= Yes, I forgone of leisure time due to informal care | G3 |
|  | If ‘Yes’ for _____(number) fewer hours per week  since _______(year) | | Continuous variable | No change | G3.1  G3.2 |
| Which activities they would perform if informal care were not needed | More paid work  No  Yes | Binary  variable | | Re-code to  0=No  1=Yes | G4 |
|  | If ‘Yes’ ___hours per week | Continuous variable | | No change | G4.1 |
|  | More unpaid work  No  Yes | Binary  variable | | Re-code to  0=No  1=Yes | G4.2 |
|  | If ‘Yes’ ___hours per week | Continuous variable | | No change | G4.3 |
|  | More leisure time  No  Yes | Binary  variable | | Re-code to  0=No  1=Yes | G4.4 |
|  | If ‘Yes’ ___hours per week | Continuous variable | | No change | G4.5 |
| Gross personal income informal caregiver* | 1. no income  2. less than 10000 KZT  3. from 10001 to 50000 KZT  4. from 50001 to 100000 KZT  5. from 100001 to 150000 KZT  6. from 150001 to 200000 KZT  7. from 200001 to 250000 KZT  8. from 250001 to 300000 KZT  9. from 300001 to 500000 KZT  10. from 500001 to 1000000 KZT  11. more than 1000000  KZT | Ordinal variable | | Re-code to  Low  Middle  High | G5 |
|  |  |  | |  |  |
| *Conversion: 1 USD = 450.99 KZT (the exchange rate on 17 June, 2023) | | | | | |

Table S2. Interview questions for exploring the burden and support needs of informal caregivers for older adults in Kazakhstan

| Interview Questions: |  |
| --- | --- |
| Care Experience | Firstly, I would like to ask you to tell me about your history.  When did you first start providing care, and how has caregiving changed over time?  Please share your experience in caring for your elderly family member, including assistance with disease management and both physical and psychological care.  Please describe the types of assistance you provide to your elderly family member.  Do you find that caregiving has become more challenging?  Have you experienced changes in their personality that affect your relationship both emotionally and practically?  Do close relatives take responsibility for the daily life of the elderly person?  Who actually helps you on difficult days? |
| Future Concerns and Plans | Based on the current caregiving needs, what concerns you about caring for the elderly in the future?  What plans do you have for caring for him/her as they age? |
| Health and Well-being | How has caregiving for your elderly family member impacted your own life in terms of family, work, and social activity?  I would like to ask you about your health (physical and mental).  How do you find time for yourself? |
| Caregiving and Support | Does the care recipient stay alone at home, and if so, why?  How have you ensured that the needs of the elderly person are met in recent years?  What needs and requirements have emerged as a result of the caregiving you provide?  Please describe the types of support you have received or would like to receive to help you take care of yourself.  Please describe the types of support you have received or would like to receive to help you care for your elderly family member.  Do you believe that individuals providing home care for the elderly should receive assistance?  In your opinion, what kind of assistance should individuals providing care receive?  What measures do you think should be taken to improve the quality and accessibility of such assistance? |
| Availability and Accessibility of healthcare and Social Care Services | Please tell me how you can contact your doctor, nurse, or social worker.  Can you call or message to ask questions and receive consultations from your doctor, nurse, or social worker?  Does a member of the primary care team contact the elderly between visits to monitor their condition?  Are consultations provided to the relatives of patients regarding caregiving and the organization of the lives of patients who have lost the ability to care for themselves?  How much attention does medical-social care pay to maintaining the mental health of those providing home care for the elderly? |
